# Multi-scale design and optimization of antibody production via flexible nets

**DOI:** 10.1016/j.csbj.2025.03.040

**Published:** 2025-04-03

**Authors:** Jorge Lázaro, Teresa Joven, Diana Széliová, Jürgen Zanghellini, Jorge Júlvez

**Affiliations:** a Department of Computer Science and Systems Engineering, University of Zaragoza, Zaragoza, Spain; b Department of Analytical Chemistry, University of Vienna, Vienna, Austria

**Keywords:** Flexible nets, Formal models, Metabolic networks, CHO cells, Antibody production

## Abstract

Antibodies are therapeutic proteins with many applications in medicine, such as treating viral infections, different types of cancer, and common diseases such as psoriasis and multiple sclerosis. Chinese Hamster Ovary (CHO) cells are the most widely used cells for antibody production due to their well-established use and favorable features. However, the current design of antibody production systems often relies on a “trial and error” approach to manipulate CHO cells. This approach is time-consuming and costly, and can lead to suboptimal process performance. The use of mathematical models has the potential to greatly accelerate and improve the design and optimization of antibody production. Starting from a systematic and formal approach, the aim is to achieve an automatic design of the whole process that allows optimal productivity to be reached. To this end, we develop mathematical models and methods for the design and optimization of antibody manufacturing systems. The mathematical models are based on Flexible Nets (FNs), a modeling formalism that accommodates uncertain parameters and nonlinear dynamics. FNs enable the development of comprehensive models that encompass both the metabolic network of CHO cells and the dynamics of the bioreactor in which the cells are cultured. Thus, by integrating macroscopic variables (e.g. dilution rate, substrate concentration, cell density, etc.) with microscopic variables (such as intracellular metabolic fluxes), our model represents a multi-scale system and facilitates global optimization.

## Highlights

• Flexible Nets combined intracellular fluxes with bioreactor dynamics for modeling.
• The model was validated, capturing key constraints like medium and uptake rates.
• A trade-off between cell growth and IgG production was detected.
• The model showed improved IgG yields by adjusting the biomass and dilution rate.
• Including uptake rates provided a framework for IgG production and medium enhancing.

## Graphical abstract

**Figure:**
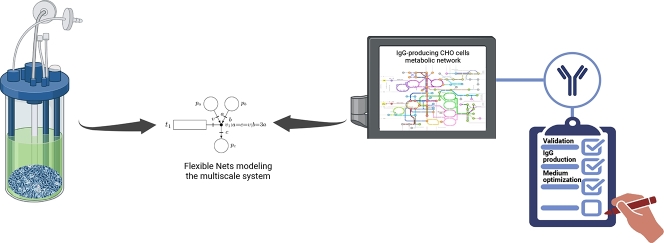

## 1 Introduction

Monoclonal antibodies are becoming increasingly important therapeutic agents in medicine. Their usefulness has been demonstrated in various applications, such as in the treatment of tumors [1], [2] and autoimmune diseases [3], [4], as well as in biosensors [5]. For an antibody drug to be effective, it must have low immunogenicity and high efficiency in its intended application [6]. To achieve these properties, antibodies require multiple post-translational modifications, proper folding, and optimal cleavage [7]. Mammalian cells are the most popular and promising production host system to effectively meet these requirements [8].

Here, we focus on Chinese Hamster Ovary (CHO) cells. CHO cells are capable of producing antibodies with glycosylation patterns similar to human serum antibodies. Although there may be some differences due to the absence of certain enzymes in CHO cells compared to the human system [9], these antibodies closely resemble endogenous human antibodies. This similarity results in low immunogenicity and increased effectiveness, making these antibodies safer for therapeutic use. Moreover, CHO cells have a lower risk of viral contamination and spreading compared to other cell lines. For these reasons, CHO cells are the preferred system for producing large amounts of monoclonal antibody drugs while maintaining high product quality [8].

Advances in technology have led to a 100-200-fold increase in antibody production in CHO cell cultures in the last 30 years [9]. However, large-scale cultures remain non-optimized due to their high cost relative to the total production of antibody drugs. The Design of Experiments (DoE) can optimize chemical production by identifying key process parameters, but it has limitations in capturing metabolic complexity and cellular responses over time. Since it relies on statistical correlations rather than mechanistic insights, in addition to being experimentally intensive, DoE can be less effective in predicting metabolic shifts [10], [11]. Computational models of bioprocesses and cell metabolism offer a promising solution by guiding researchers in experimental design. By shifting from trial-and-error to rational bioprocess and cell line optimization, this approach can reduce investment costs and improve antibody production efficiency [12], [13], [14], [15].

The modeling of metabolic networks can be approached with several key methods. Constraint-based model methods, such as Flux Balance Analysis (FBA) [16], use stoichiometry and optimization to predict cell behavior under steady-state assumptions, making them effective for genome-scale applications. Kinetic models [17] incorporate enzyme kinetics to capture transient time-dependent changes in metabolic fluxes, but often require extensive data. Metabolic Flux Analysis (MFA) [18] quantifies fluxes through measured uptake and secretion rates in simpler systems. Hybrid models, like dynamic FBA (dFBA) [19], combine constraint-based models with dynamic elements to simulate changes over time. On the other hand, Petri nets [20] offer a graphical approach that enables the representation of regulatory and probabilistic elements.

Genome-scale metabolic models (GEMs) [21] are mathematical depictions of an organism's metabolism. They encode a comprehensive network of mass-balanced connections among the metabolites of the organism, based on gene-protein-reaction associations derived from genome annotation and experimental data. Although these models are not entirely accurate, many have been proven reliable enough to aid in decision making for wet lab experiments [22], [23]. GEMs are inherently limited in their ability to capture multiscale variables, such as those arising from interactions between a bioreactor environment and a cell culture, because they focus primarily on intracellular metabolic reactions defined by stoichiometric constraints. GEMs operate at the cellular level, typically assuming a static, controlled environment without accounting for external variables like nutrient supply rate, pH shifts, product toxicity, biomass generation or other dynamic conditions that occur in a bioreactor [21]. These external factors can significantly impact cellular metabolism by altering reaction kinetics, product formation, and metabolic flux distributions.

Although the aforementioned approaches are valuable tools for modeling many real systems, they lack sufficient modeling power or analytical methods to address large multi-scale systems with uncertainties and nonlinear dynamics. Thus, alternative modeling frameworks and computational methods are necessary.

Flexible Nets (FNs) [24] is a modeling formalism that, in addition to representing the complete set of intracellular fluxes within a GEM, can incorporate easily bioreactor dynamics, including macroscopic variables such as dilution rate, biomass growth, and concentration of metabolites in the medium and in the tank. FNs offer a straightforward and intuitive graphical representation, and enable the modeling of regulation processes such as reaction activation or inhibition based on metabolite concentration [25]. Additionally, FNs can approximate nonlinear dynamics via piecewise linear functions and model uncertainty, enhancing their versatility as a modeling approach.

To demonstrate that FNs is a suitable formalism to model and optimize production by CHO cells, we model antibody production in a bioreactor operated in continuous culture mode, i.e., nutrients are continuously supplied to the tank at a specific dilution rate. Simultaneously, cells, cell products, and waste products are removed from the tank via the effluent, also regulated by the dilution rate. Although bioreactors often operate in batch and fed-batch modes for many biotechnological processes, there is a growing expectation that continuous cultures will become more prevalent [26], [27]. Thus, our current modeling assumes a static exchange profile (given by measurements) of the cell with its environment, rather than dynamic changes over time. This allows us to investigate the trade-offs between growth and production using a novel methodology that has not been applied in this context before. Moreover, FN provides a flexible foundation for future work, where time-resolved exchange fluxes or other data can be seamlessly integrated, highlighting its advantages over dFBA for scalable and data-rich analyses.

In addition to validating and evaluating the model's performance, we also considered relevant aspects related to the antibody production optimization. One of these aspects is media design. Since not all amino acids in the medium have the same cost, and some may be present in excess beyond the requirements for antibody production, optimizing amino acid concentrations is essential.

In metabolic modeling, a phenotype represents a specific metabolic behavior defined by the flux distribution across reactions under given constraints. Different conditions, such as nutrient availability or gene knockouts, shape distinct phenotypes by altering metabolic fluxes. In our case, when producing a particular antibody, different phenotypes can be reached depending on the dilution rate and the biomass concentration in a bioreactor, each with distinct amino acid consumption patterns. Identifying the minimum amino acid requirements for each phenotype would help avoid resource waste and reduce production costs.

There are multiple approaches to develop an optimized medium, though many are based on empirical observations [28], [29]. In this study, we utilize FNs to predict the minimum concentration of each amino acid in the medium needed to achieve a specific IgG concentration, or, in other words, a desired phenotype. While our product of interest is the antibody IgG, this approach can be applied to the production of other metabolites.

## 2 Materials and methods

### 2.1 Flexible nets

This section introduces Flexible Nets (FNs) [24], a modeling formalism inspired by Petri Nets [30], [31]. FNs can model effectively the interplay between the state and the processes of dynamic systems through two interconnected nets: the event net and the intensity net. While the event net defines how processes influence state variables, the intensity net determines how these variables regulate processes' rates [32].

Both the event and the intensity nets are tripartite graphs, comprising places (associated with metabolites) depicted as circles (set *P*), transitions (linked with reactions) depicted as rectangles (set *T*), and handlers depicted as dots. There are two types of handlers: event handlers (set *V*), which in the context of metabolic networks, trigger changes in metabolite concentrations when a reaction occurs, and intensity handlers (set *S*) which determine the influence of metabolite concentrations on reaction rates.

Let us consider a simple system to exemplify some modeling capabilities of FNs. Hemoglobin is a tetrameric protein responsible for oxygen transport in the blood, consisting of two alpha and two beta subunits. It can bind a variable number of oxygen molecules, ranging from 1 to 4, depending on oxygen availability. Each subunit can independently bind an oxygen molecule. Although hemoglobin is most efficient when fully saturated with four oxygen molecules, it remains functional across intermediate states, allowing it to release oxygen to tissues based on their specific needs [33].

If one molecule of hemoglobin binds two molecules of oxygen, the process results in two molecules of oxygen bound to one molecule of hemoglobin. Such a reaction can be represented as:

$$R_{1}:M_{a}+2M_{b}\to M_{c}$$

where $R_{1}$ has two reactants, $M_{a}$ (hemoglobin) and $M_{b}$ (oxygen), and one product, $M_{c}$ (oxygen-bound hemoglobin).

**Fig. 1:**
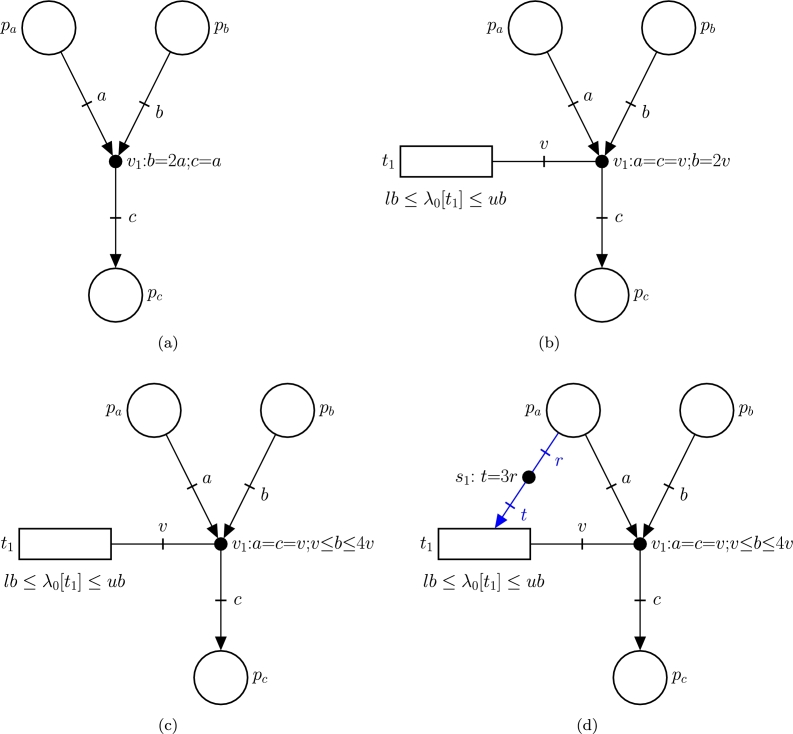
(a) Event net modeling the stoichiometry of reaction *R*_1_:*A* + 2*B* → *C*. (b) Event net modeling the reaction *R*_1_:*A* + 2*B* → *C* with rate *λ*_0_[*t*_1_] constrained in the interval [*lb*,*ub*]. (c) Event net modeling the reaction *R*_1_:*A* + *nB* → *C* where *n* is the uncertain stoichiometric coefficient of *p*_*b*_. (d) Flexible Net modeling the reaction *R*_1_:*A* + *nB* → *C* with rate equal to *λ*_0_[*t*_1_] plus 3 times the concentration of *M*_*a*_. Here, the event net is represented by black arcs and edges, and the intensity net with blue arcs and edges.

The event net in Fig. 1a, which models reaction $R_{1}$, has three places, $P=\{p_{a},p_{b},p_{c}\}$ which are associated with $M_{a}$, $M_{b}$ and $M_{c}$ and one event handler $V=\{v_{1}\}$. The labels of the arcs, *a*, *b*, and *c*, represent the number of hemoglobin molecules consumed, the number of oxygen molecules consumed, and the number of oxygen-bound hemoglobin molecules produced by reaction R1, respectively. Since one hemoglobin molecule binds to two oxygen molecules, *b* must be twice *a*, i.e. b=2a. Additionally, because one oxygen-bound hemoglobin molecule is produced for each hemoglobin molecule, *c* must be equal to *a*, i.e. c=a. This way, the equations $v_{1}:b=2a;c=a$ associated with event handler $v_{1}$ define the relationships between the number of molecules consumed and produced by a reaction.

Notice that the net in Fig. 1a does not contain any dynamic information, i.e. the reaction rate is undefined. Such a rate can be modeled by adding the transition $t_{1}$, see Fig. 1b. Transition $t_{1}$ is connected to $v_{1}$ through an edge with label *v* which represents the number of occurrences of the reaction. Thus, the equations a=c=v and b=2v associated with the event handler $v_{1}$ imply that one occurrence of $R_{1}$ (v=1) consumes one molecule of $M_{a}$ (a=1), two molecules of $M_{b}$ (b=2) and produces one molecule of $M_{c}$ (c=1). The reaction rate of $R_{1}$ is given by the variable $\lambda_{0}[t_{1}]$ which is associated with $t_{1}$. In this particular example, $\lambda_{0}[t_{1}]$ can be any real value in the interval [lb,ub] (see the inequalities associated with $t_{1}$).

The net in Fig. 1c accounts for the fact that the number of oxygen molecules that can bind one molecule of hemoglobin is not a fixed value, but lies in the interval [1,4]. In this case, the reaction can be expressed as:

(1) $$R_{1}:M_{a}+nM_{b}\to M_{c}$$

where *n* is an uncertain stoichiometric coefficient, that is, *n* units of $M_{b}$ in the interval [1,4] are consumed per each unit of $M_{a}$ to produce one unit of $M_{c}$. Thus, the number of molecules of $M_{b}$ consumed per each occurrence of $R_{1}$ is an uncertain value in the interval [1,4]. In terms of fluxes (or rates), Equation (1) implies that the flux at which $M_{b}$ is consumed is *n* times the flux at which $M_{a}$ is consumed, and the flux at which $M_{a}$ is consumed is equal to the flux at which $M_{c}$ is produced.

Intensity nets can be used to model how reaction rates depend on the concentration of metabolites. In Fig. 1d, the intensity net contains an intensity handler $s_{1}$, an edge from $p_{a}$ to $s_{1}$, and an arc from $s_{1}$ to $t_{1}$. The equation t=3r associated with $s_{1}$ means that the rate, *t*, produced by $M_{a}$ in $R_{1}$ is equal to 3 times the concentration of $M_{a}$, *r*. Thus, $R_{1}$ is a first order reaction. This rate is added to $\lambda_{0}[t_{1}]$, which is in [lb,ub], to obtain the final reaction rate λ[t]. Similarly to event handlers, inequalities can be associated with intensity handlers to model uncertainties, e.g. 2r≤t≤4r would mean that the rate produced is between 2 and 4 times the concentration of $M_{a}$.

The reaction rate of $R_{1}$ in Fig. 1d is linear with respect to the concentration of $M_{a}$. In order to account for nonlinear dynamics, piecewise linear functions can be associated with the intensity handlers. For instance, if the piecewise linear function:

(2) $$s_{1}:\;\left\{ \begin{array}{c} t=3r\text{ if }r\leq 5\\ t=15\text{ if }r>5 \end{array}\right.$$

is associated with $s_{1}$, then the rate produced by $M_{a}$ will be equal to 3 times its concentration if such a concentration is equal or lower than a given threshold 5, otherwise the rate produced will not be proportional to the concentration but constant and equal to 15.

In most GEMs, reactions are described in terms of their stoichiometry (without uncertain coefficients) and flux bounds that are independent on metabolite concentrations. Thus, such reactions can be modeled straightforwardly through event nets similar to the one in Fig. 1c.

### 2.2 Chinese Hamster Ovary cell model (iCHOv1)

#### 2.2.1 iCHOv1 features

The iCHOv1 model from BiGG Models [34] is a comprehensive representation of the metabolic network of Chinese Hamster Ovary (CHO) cells, widely used in biopharmaceutical production [13]. It accounts for 4456 metabolites, 6663 reactions, and 1766 genes, and integrates extensive biochemical data, covering central carbon metabolism, amino acid and nucleotide biosynthesis, lipid metabolism, and energy production. The model includes Gene-Protein-Reaction (GPR) associations, linking specific genes to the enzymes and metabolic reactions they encode. It also has an artificial biomass composition reaction, precise reaction stoichiometry for mass and energy balance, and accounts for the following cellular compartments: cytosol, mitochondria, endoplasmic reticulum, and extracellular space.

#### 2.2.2 IgG antibody synthesis reaction

Immunoglobulin G (IgG) is a versatile and abundant antibody crucial for immune response. Structurally, IgG is Y-shaped, with two heavy chains (Hc) and two light chains (Lc), and has two antigen-binding sites at the Y-tips for recognizing pathogens [35], [36].

The iCHOv1 model does not contain the reactions required to produce IgG. To enable antibody synthesis, we added these reactions to the model. Given that IgG is a protein, amino acids are sequentially integrated in the polypeptide chain until one IgG subunit, $IgG_{subunit}$, is completed (see reaction $R_{1}$ below), as defined in [37]. Afterwards, two antibody subunits must assemble to produce the functional IgG (see $R_{2}$). To facilitate antibody secretion, we also included an exchange reaction (see $R_{3}$). The complete list of added reactions is as follows:

$$R_{1}:\;39Ala\;\text{+}\;14Arg\;\text{+}\;...\;\text{+}\;1324GTP\;\text{+}\;1326H_{2}O\;\;\to IgG_{subunit}\;\text{+}\;...\;\text{+}\;1326H\;R_{2}:\;2IgG_{subunit}\to IgG\;R_{3}:\;IgG\to \emptyset$$

**Fig. 2:**
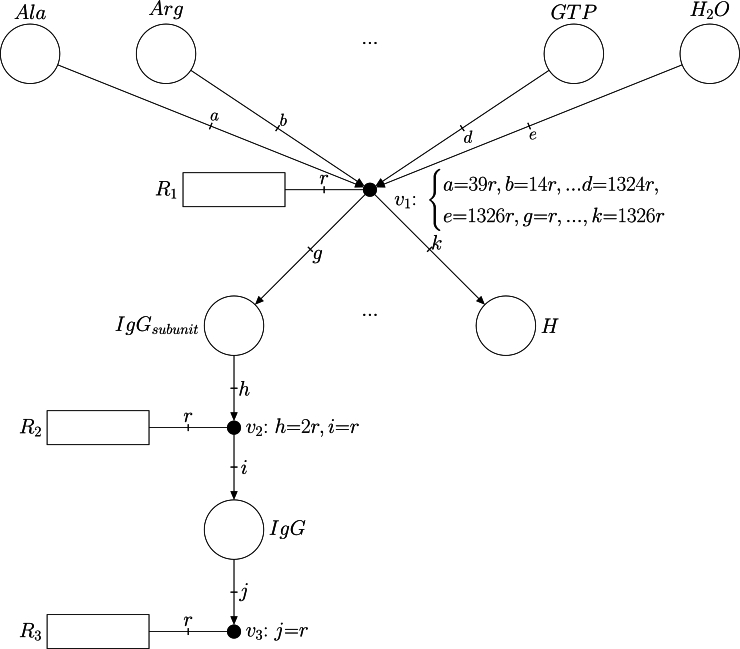
FN modeling the reactions that produce IgG in the iCHOv1 model. Note that in reaction *R*_1_, only some of the involved amino acids are represented (see Table 3 for the comprehensive list of stoichiometric coefficients).

An FN graphical representation of these reactions is shown in Fig. 2. The labels a, b, c, etc. model the stoichiometry of each reaction. For instance, a=39r in reaction $R_{1}$ means that 39 moles of the amino acid *Ala* react to produce a mole of the $IgG_{subunit}$. The complete reaction containing all the stoichiometric coefficients is reported in Table 3 (see Annex).

### 2.3 Modeling a continuous culture with FNs

**Fig. 3:**
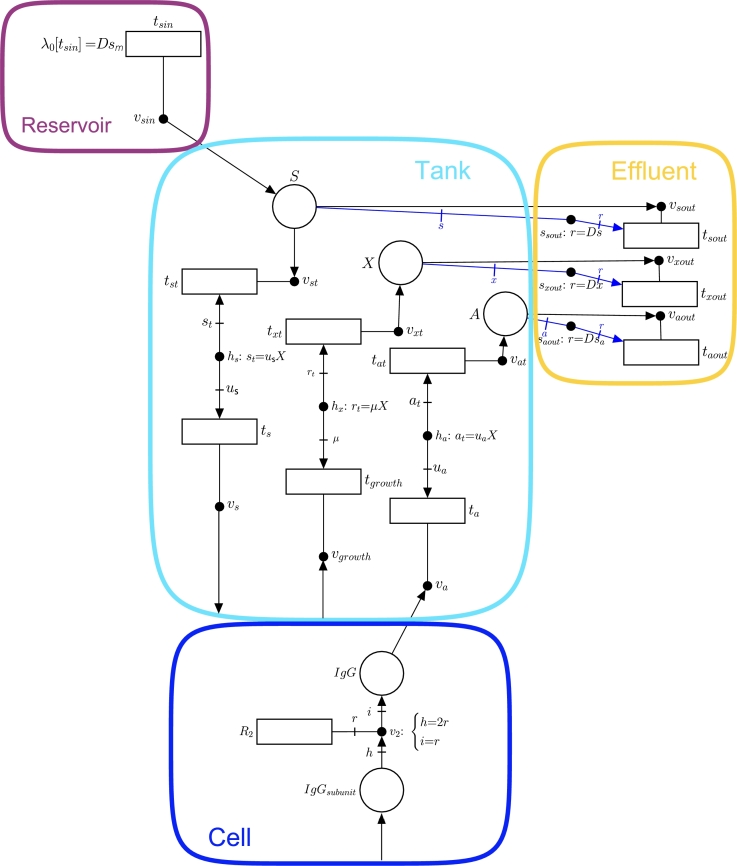
Bioreactor consisting of three compartments: reservoir, tank and effluent. The blue rectangle “Cell” at the bottom contains the whole metabolic network of iCHOv1.

The bioreactor used for the continuous culture consists of three compartments: the reservoir, the tank and the effluent. Fig. 3 sketches an FN model that integrates the microscopic variables of the model, i.e., the fluxes of the reactions in the metabolic network of a CHO cell, with the macroscopic variables of the bioreactor, see Table 1.

**Table 1:** Macroscopic variables involved in the bioreactor dynamics.

Macroscopic variables	Units	Description
D	*h*^−1^	Dilution rate
*s*_*m*_	*mM*	Nutrient concentration in medium
*s*_*t*_	*mM*	Nutrient concentration in tank
X	*gDWL*^−1^	Biomass concentration
*a*_*t*_	*mM*	Antibody concentration in tank

For a better understanding of Fig. 3, the step-by-step model development process is discussed below.

1. **Add the necessary reactions to the GEM to produce IgG:** The GEM iCHOv1 is extended to accommodate the reactions detailed in Section 2.2.2 for IgG production. The cell compartment in Fig. 3 contains the whole iCHOv1 GEM together with these reactions (for clarity, only reaction $R_{2}$ for antibody production is depicted).
  Before discussing the next steps, it must be noted that the evolution of the concentration of a given nutrient, *s*, in a bioreactor is determined by:
  (3) $$\frac{ds_{t}}{dt}=(s_{m}-s_{t})D-u_{s}X$$
  where $s_{m}$ is the concentration of nutrient *s* in the medium, $s_{t}$ is the concentration of the same nutrient in the tank, *D* is the dilution rate, $u_{s}$ is the uptake rate of the nutrient by the cell, and *X* is the biomass concentration (see Table 1 for a description of the macroscopic variables).
  Note that the terms in Equation (3) have a negative contribution if the metabolite is removed from the tank, and a positive contribution if the metabolite goes from a certain compartment into the tank. Steps 2, 3 and 4 explain how each of these terms is modeled by FNs.
2. **Model nutrients' fluxes from the medium to the tank (**$Ds_{m}$**):** A given nutrient *s*, with concentration $s_{m}$ in the medium, is supplied from the reservoir to the tank at a dilution rate *D*, i.e. the flux of *s* from the reservoir to the tank is $Ds_{m}$ (see Equation (3)). This flux is modeled by the transition $t_{sin}$ in Fig. 4(a) whose intensity is $\lambda_{0}[t_{sin}]=Ds_{m}$. Note that, although only one nutrient is depicted in Fig. 3, the model accounts for all the nutrients in Table 5.

**Fig. 4:**
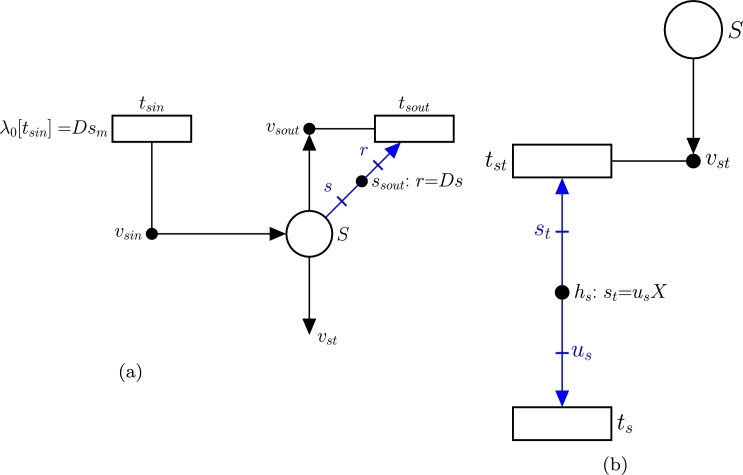
(a) Subnet modeling the relationship between the reservoir, the nutrient in the tank and the effluent in the bioreactor. (b) Subnet modeling nutrient uptake.
3. **Model the nutrient uptake from the tank (**$-u_{s}X$**):** Once a nutrient *s* is in the tank (such a nutrient is modeled by place *S*), it can be either transported into the cell or expelled to the effluent. The former process corresponds with the term $-u_{s}X$ and is modeled by Fig. 4(b). Such transport of *s* from the tank to a single cell is modeled by transition $t_{s}$, which matches with the exchange reaction of *s* in the iCHOv1 model. This exchange reaction will have a metabolic flux $u_{s}$ indicating the rate of consumption of metabolite *s* by the cell. The key element that gathers the microscopic (intracellular fluxes of iCHOv1) and macroscopic (bioreactor dynamics) variables and allows for the development of a multi-scale model is the intensity handler $h_{s}$. The equation associated with $h_{s}$ scales the exchange flux of metabolite *s* by the biomass concentration *X*, i.e. $s_{t}=u_{s}X$ (see Equation (3)), to obtain the overall flux of *s*, $\lambda [t_{st}]$, consumed by the cell culture.
4. **Model nutrients' fluxes from the bioreactor to the effluent (**$-Ds_{t}$**):** As mentioned in the previous step, a certain amount of the metabolite modeled by place *S* will be carried by the effluent, preventing it from being consumed by cells. This process is depicted in Fig. 4(a), specifically by $t_{sout}$ and the elements connected to it ($v_{sout}$ and $s_{sout}$). The rate at which this process occurs is defined by the intensity handler $s_{sout}$ (see Equation (3)).
5. **Model the biomass in the bioreactor:** The biomass evolution in the bioreactor is given by:
  (4) $$\frac{dX}{dt}=(\mu -D)X$$
  where *μ* is the specific growth rate of the cell, which coincides with the flux of the biomass reaction of the iCHOv1 model, *D* is the dilution rate, and *X* is the biomass concentration in the bioreactor.
  The first part of Equation (4), *μX*, is modeled with FNs in the same way as in step 3, with a slight difference. In this case, the term is positive because the growth rate *produces* biomass in the tank. In this part of the model, the intensity handler $h_{x}$ acts as the interface between the microscopic and macroscopic parts of the model (Fig. 5(a)).

**Fig. 5:**
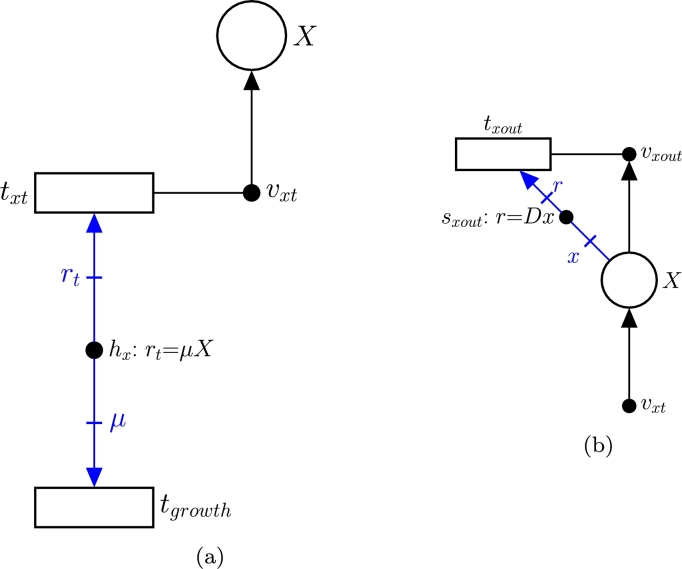
(a) Subnet modeling biomass production. (b) Subnet modeling the output flux of *X*.
  The second part of the Equation (4), −DX, models how cells are removed from the tank towards the effluent ($t_{xout}$) at the rate indicated by Equation (4) (see Fig. 5(b)).
6. **Add the IgG antibody production:** The concentration of antibodies in the bioreactor is determined by:
  (5) $$\frac{da_{t}}{dt}=u_{a}X-Da_{t}$$
  Equation (5) is closely related to Equation (3). The first part consists of $u_{a}X$, where $u_{a}$ is the flux of the antibody exchange reaction from the modified iCHOv1 model ($R_{3}$ in Fig. 2). This cellular-level reaction is connected to the macroscopic variables through the intensity handler $h_{a}$ (Fig. 6(a)). Note that the term is positive, since the antibody is produced and secreted from cell to the tank.

**Fig. 6:**
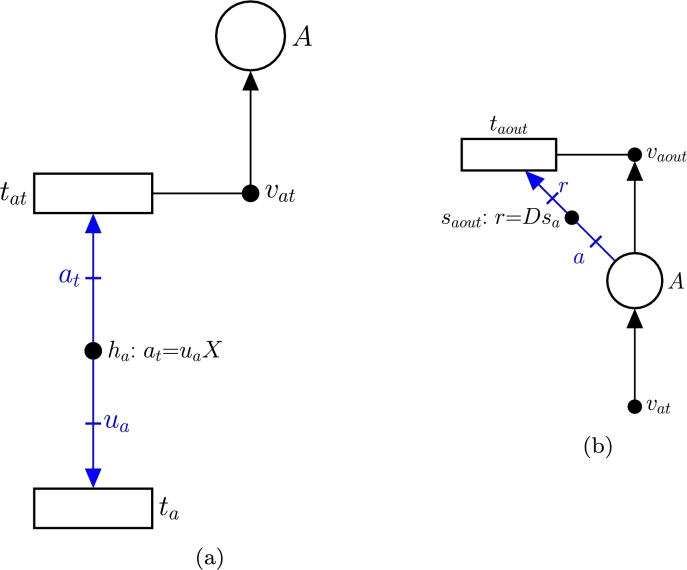
(a) Subnet modeling the production of antibodies. (b) Subnet modeling the flux of antibodies from the bioreactor to the effluent.
  The second term of Equation (5), $Da_{t}$, is the rate at which the antibody leaves the tank through the effluent as captured in $s_{aout}$ (Fig. 6(b)).

Notice that Equations (3), (4) and (5) are all equal to zero since we assume the steady-state condition, typically reached in the continuous operational mode of a bioreactor. Thus, in order to analyze and optimize the resulting FN, a set of mathematical constraints that necessarily hold at steady-state will be derived [32]. These constraints, along with an appropriate objective function, e.g. the maximization of the flux of the antibody exchange reaction, will form a programming problem that can be used to make various predictions.

### 2.4 Implementation of the optimization procedure

The reactions related to the synthesis of antibodies were added to the iCHOv1 that was obtained from the BiGG Models database, (http://bigg.ucsd.edu/models/iCHOv1). This modified model was converted into a Flexible Net (FN) by utilizing the open-source Python package fnyzer (Flexible Nets analYZER). FNs allowed us to model a multiscale system that integrates the metabolic network of CHO cells and the dynamics of the bioreactor. Then, the addition of flux bounds, steady state conditions and an objective function (in this case, maximizing the IgG antibodies production) resulted in a linear programming problem (LPP) whose solution represented a theoretical maximum for IgG production. The optimization software used by fnyzer to solve the resulting LPP was CPLEX [38].

The modifications in the iCHOv1 model were performed using the 0.27.0 version of COBRApy Python package [39]. All the simulations and the analyses were conducted by fnyzer 1.3.9 [40] in combination with the CPLEX solver. The developed scripts are available at: https://github.com/jlazaroibanezz/IgG_CHO_FNs.

Simulations were run on a workstation equipped with an 11th Gen Intel(R) Core(TM) i7-1165G7 processor (2.80 GHz, 4 cores) and 15 GB of RAM. The system was running Ubuntu 22.04.3 with Linux kernel version 6.8.0. One optimization of the system takes less than 30 seconds.

## 3 Results

### 3.1 Model validation

To evaluate our model's predictive capacity, we compared its predicted IgG production with experimentally reported values from [15]. This comparison was based on two datasets, each containing experimental uptake rates of medium metabolites (in mmol $g_{DW}^{-1}$ $h^{-1}$) and the growth rate of the cell culture ($h^{-1}$).

The first dataset, from [41], represents measurements taken during the late exponential phase of cell culture growth (Late Exponential phase dataset). The second dataset, from [12], includes measurements from a different cell culture during its exponential phase (HP dataset). Both datasets correspond with a fed-batch operation mode, in which nutrients are added in a controlled manner following an initial growth phase (batch phase). For additional details on these datasets, see Annex (Table 4).

The equations describing the bioreactor dynamics (Equations (3), (4) and (5)) specifically model the dynamics of a continuous bioreactor. We used the data of the exponential phase of the fed-batch datasets as a good approximation for our model parameters. Our continuous culture FN model aligns with FBA conditions, as both maintain constant metabolite concentrations and fluxes, ensuring a balanced metabolic network in steady-state. The growth rate in a chemostat, constrained by the dilution rate, mirrors the fixed exchange fluxes in FBA, making our optimization process a relevant approach for studying metabolism under steady-state conditions [41], [15]. Previous studies have used genome-scale metabolic models to analyze CHO cell metabolism in fed-batch cultures, applying FBA and FVA under steady-state assumptions [12], [41]. Similarly, we integrated experimental uptake rates from fed-batch operations into our FN model and compared predicted and experimental IgG production (Table 2).

**Table 2:** Antibody production in *mmol*$g_{DW}^{-1}$*h*^−1^. The row “Model” reports the model predictions for the two experimental datasets assessed (Late Exponential phase and HP). The row “Experimental” contains the antibody production in [41] for the Late Exponential phase dataset, and in [12] for the HP dataset.

	HP	Late Exp phase
Experimental	2.02 ⋅ 10^−5^	2.44 ⋅ 10^−5^
Model	2.04 ⋅ 10^−5^	3.205 ⋅ 10^−5^
Relative error	9.9 ⋅ 10^−3^	0.3

The final FN-based model integrates the following constraints: 1) the medium formulation, 2) experimental upper bound flux constraints for the uptake rates of each amino acid in the cell culture retrieved from [41], [12], 3) the equations modeling the bioreactor dynamics, and 4) the mass balance and stoichiometric constraints inherent in iCHOv1. Once the model is transformed into an FN by means of the fnyzer Python package [40], we impose the steady-state condition and set as objective function the maximization of the flux of the antibody synthesis reaction ($R_{3}$ in Fig. 2). This results in a null flux for the growth rate. Conversely, when the objective function is the maximization of the growth rate, the flux of the antibody synthesis reaction becomes zero. This implies that the available medium resources are entirely allocated either to antibody synthesis or to growth, depending on the selected objective function. Constraining the growth rate with the experimental values provided in the “Experimental growth rate” row from Table 4 [12], [41] overcomes the problem.

By imposing the steady-state condition and maximizing the IgG synthesis under the aforementioned constraints, we predicted the values reported in Table 2 (row ‘Model’). These predicted values were then compared to the experimentally measured IgG synthesis reaction fluxes (Experimental). The relative error was computed as follows:

(6) $$Relative\;Error=\frac{\left|Model-Experimental\right|}{Experimental}$$

Our model tends to overestimate the IgG production in all cases. However, the prediction improves when the model is enriched with the experimental data from the HP dataset. Although the relative error for the Late Exponential phase dataset is higher, it is not significant, and the experimental and predicted IgG production values remain within the same order of magnitude (see Table 2).

### 3.2 IgG production optimization

After evaluating the predictive capacity of our model, we further investigated how the parameters of the macroscopic variables could influence the production of IgG in the cell culture. We selected typical intervals for the dilution rate and cell density observed in CHO cell cultures and focused on maximizing the IgG synthesis reaction flux while fixing the growth rate to the sampled dilution rates, as described in Equation (4) (equal to zero in steady-state).

**Fig. 7:**
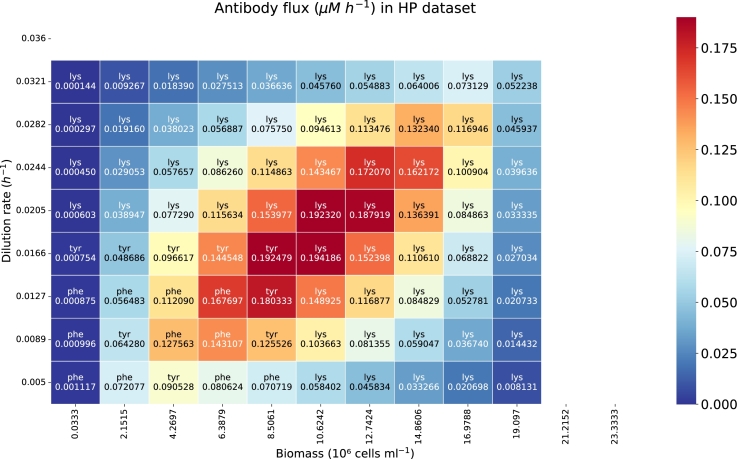
Heatmap showing the IgG synthesis reaction flux (*μM h*^−1^) as a function of the dilution rate (*h*^−1^) and biomass (10^6^ cells *ml*^−1^). The abbreviation of the limiting amino acid is indicated for each biomass and dilution rate sampled. The implemented data corresponds with the HP dataset.

The heatmap in Fig. 7 shows the flux of IgG synthesis, measured in *μ*M $h^{-1}$, as a function of dilution rate ($h^{-1}$) and biomass concentration (10^6^ cells $ml^{-1}$) using data from the HP dataset. The peak IgG production is observed in the central region of the heatmap, with the highest flux value of 0.194 *μ*M $h^{-1}$ occurring at a dilution rate of 0.0166 $h^{-1}$ and a biomass concentration of 10.624 $\cdot 10^{6}$ cells $ml^{-1}$. Blank rows and columns represent regions where no feasible optimization solution exists.

**Fig. 8:**
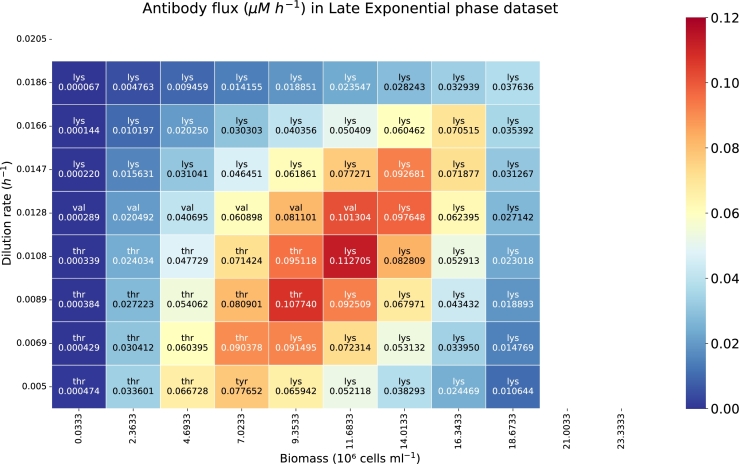
Heatmap with the IgG synthesis reaction flux (*μM h*^−1^) as a function of the dilution rate (*h*^−1^) and biomass (10^6^ cells *ml*^−1^). The abbreviation of the limiting amino acid is indicated for each biomass and dilution rate sampled. Here we implemented the data from the Late Exponential dataset.

In comparison, Fig. 8 shows lower overall IgG production rates when using parameters derived from the Late Exponential phase dataset. This suggests that the Late Exponential phase data impose stricter constraints, which limit the IgG reaction flux more significantly than the parameters derived from the HP dataset.

An important consideration when working with culture media is identifying the limiting metabolite. To determine which metabolite is limiting in each scenario, we implemented the following strategy:

1. Fix the IgG synthesis reaction flux to the value obtained when maximized.
2. Maximize and minimize the fluxes of the exchange reactions for each amino acid one at a time.
3. Compute the difference between the maximum and the minimum exchange fluxes for each amino acid.
4. The amino acid whose difference between maximum and minimum fluxes for its exchange reaction is zero will be the limiting metabolite.

In other words, a metabolite is said to be limiting when the maximum and minimum possible fluxes of its exchange reaction are equal. Consequently, even a subtle change in the flux of the exchange reaction of the limiting metabolite can significantly impact IgG production.

The results for the HP dataset (Fig. 7) show that, at low dilution rates and cell densities, the limiting metabolite varies between tyrosine (Tyr) and phenylalanine (Phe). However, as the dilution rate and cell density increase, lysine (Lys) becomes the limiting metabolite.

For the Late Exponential phase dataset (Fig. 8), threonine (Thr), tyrosine (Tyr), and valine (Val) are limiting at low dilution rates and cell densities, but lysine (Lys) becomes limiting as these parameters increase.

### 3.3 Assessing medium uncertainty

The composition of a commercial medium may vary between batches. This can occur due to raw material variability or differences in the manufacturing process. Such batch-to-batch differences can impact cell growth and experimental outcomes, especially for sensitive cultures. To assess such an impact, the concentration of each metabolite in the medium was assumed to have a given degree of uncertainty, while the overall concentration of the medium was kept constant.

In particular, to model uncertainty in the medium, the concentration of each nutrient in the medium was not fixed to the particular value *c* provided in Table 5, but was allowed to take any value in the interval [c⋅(1−u),c⋅(1+u)] where *u* is the degree of uncertainty. Notice that this can be easily accommodated in the FN model by replacing equalities by inequalities.

**Fig. 9:**
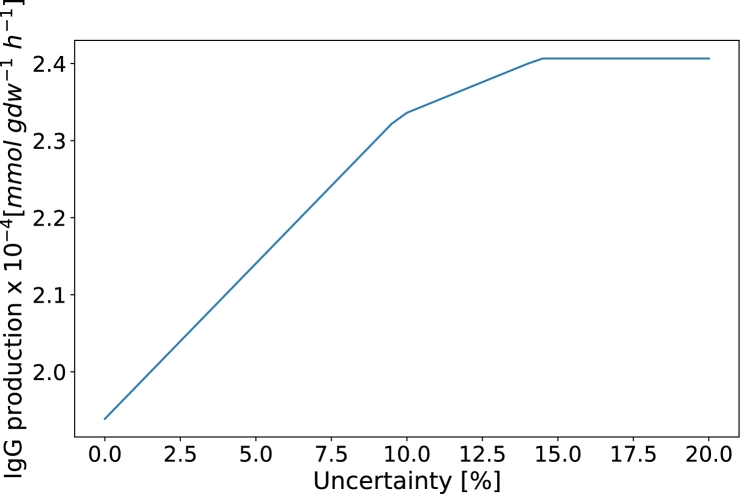
IgG production as a function of the percentage of uncertainty incorporated in the medium nutrients.

By applying the steady-state condition and optimizing the Linear Programming Problem (LPP) with an objective function, specifically the IgG synthesis reaction, the theoretical maximum IgG production was computed for values of uncertainty ranging from 0% to 25%, see Fig. 9.

It can be seen that for low degrees of uncertainty, the production increases linearly. This is because higher uncertainty allows for more flexibility in optimizing nutrient concentrations, leading to higher yields. However, when the uncertainty reaches 10%, the increase starts to slow down, and from 15%, IgG production remains constant. This behavior is due to the uptake rate limitations of individual nutrients by the cells.

### 3.4 Medium optimization

Another application of the developed model is medium optimization. We determined which nutrients are present in excess and identified the minimum concentrations required to sustain a certain level of IgG production. In particular, we focused on the following optimization objectives: 1) economic medium minimization, and 2) minimization of the number of nutrients in the medium.

#### 3.4.1 Economic medium minimization

For the economic medium minimization, we selected the dilution rate and biomass concentration at which the maximum IgG synthesis reaction flux is achieved, as shown in Fig. 7, Fig. 8. We fixed this value and minimized the weighted sum of the intensities of the incoming transitions to the tank ($t_{sin}$ in Fig. 3), with each nutrient weighted according to its market cost as listed in the US Pharmacopeia [42]. This is expressed as:

(7) $$\text{min}\sum_{s\in Nut}k_{s}\cdot \lambda [t_{sin}]$$

where *Nut* is the set of nutrients in the medium (see Table 5), $k_{s}$ is the weight based on the market cost of nutrient *s*, and $\lambda [t_{sin}]$ is the flux entering the tank from the reservoir. This optimization calculates the required concentration of each nutrient entering the tank from the reservoir compartment. We conducted this analysis both for the HP and the Late Exponential phase datasets, each corresponding to the maximum flux of the IgG synthesis reaction in each dataset.

**Fig. 10:**
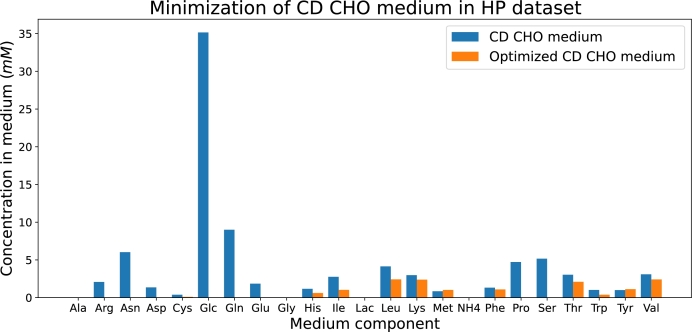
Bar plot representing the concentrations of each nutrient in the original CD CHO medium (blue bars) and in the optimized medium according to the FN-based model enriched with experimental data from the HP dataset (orange bars).

The optimization for the HP dataset produced the results shown in Fig. 10. The CD CHO medium (see Table 5), typically used for CHO cell cultures, appears to contain all nutrients in excess except for methionine (Met) and tyrosine (Tyr). According to our integrated FN model, adjusting nutrient concentrations to the levels represented by the orange bars would not affect the IgG production levels while reducing the overall medium cost.

**Fig. 11:**
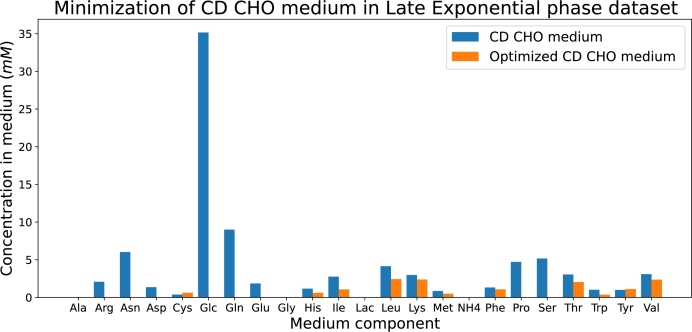
Bar plot that represents the concentrations of each nutrient in the original CD CHO medium (blue bars) and in the optimized medium according to the FN-based model enriched with experimental data from the Late Exponential phase dataset (orange bars).

We performed the same optimization generating Fig. 11, with the FN model including the parameters from the Late Exponential phase dataset.

#### 3.4.2 Minimization of the number of nutrients in the medium

This section aims to minimize the number of nutrients in the medium rather than its economic cost. To achieve this goal, we first set a null flux from the reservoir to the tank, i.e. $\lambda [t_{sin}]=0$, to each nutrient individually, this is equivalent to removing the nutrient from the medium, while keeping the biomass and dilution rate fixed at the values that produce the maximum IgG synthesis flux. Then, antibody production is maximized subject to the previous constraints in each dataset. Nutrients in the media were classified depending on their effect in IgG production:

• **Indispensable nutrients**: Nutrients whose removal from the medium results in a null antibody production.
• **Modulatory nutrients**: Nutrients whose removal reduces antibody production but does not stop it completely.
• **Redundant nutrients**: Nutrients whose removal causes no change in antibody production.

The simulations identified nine indispensable nutrients: histidine (His), isoleucine (Ile), leucine (Leu), lysine (Lys), methionine (Met), phenylalanine (Phe), threonine (Thr), tryptophan (Trp), and valine (Val); two modulatory nutrients: cysteine (Cys) and tyrosine (Tyr); and eight redundant nutrients: arginine (Arg), asparagine (Asn), aspartate (Asp), glucose (Glc), glutamine (Gln), glutamate (Glu), proline (Pro), and serine (Ser).

Additionally, we minimized the sum of all $\lambda [t_{sin}]$ values from the reservoir to the tank after having fixed the antibody production to the maximum obtained in Section 3.2:

(8) $$\text{min}\sum_{s\in Nut}\lambda [t_{sin}]$$

This optimization revealed that the maximum antibody production can still be achieved when the concentrations of all the redundant nutrients are simultaneously set to zero. Consequently, the minimum number of nutrients required to sustain the maximum IgG production along with the associated biomass is 11, i.e., the number of the indispensable nutrients plus the number of the modulatory nutrients.

**Fig. 12:**
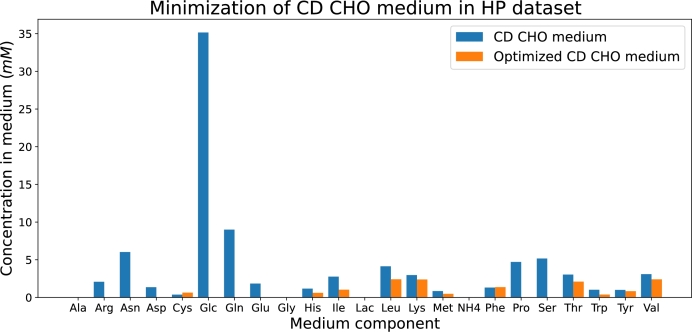
Bar plot showing the concentrations of each nutrient in the original CD CHO medium (blue bars) and in the optimized medium according to the FN-based model enriched with experimental data from the HP phase dataset (orange bars). In this case the minimization of the medium was performed focusing on the number of nutrients utilized.

Optimizing the model with Equation (8) as objective function allowed us to compute the minimum sum of fluxes of each nutrient necessary to achieve the fixed IgG production. The obtained fluxes were the same as in the previous section for the Late Exponential phase dataset, see Fig. 11. However, there were slight differences between the economic and number of nutrients minimizations when using the HP dataset, see Fig. 12. A comparison of Fig. 10, Fig. 12 shows that the economically minimized medium contains less cysteine and phenylalanine but more methionine and tyrosine compared to the medium with minimized number of nutrients. This difference is likely due to tyrosine's relatively lower cost compared to phenylalanine. Both amino acids are among the three aromatic amino acids, sharing similar structures and roles in cellular metabolism and IgG production. As a result, substituting a specific quantity of phenylalanine with tyrosine can help reduce overall costs.

## 4 Discussion

The computational model predicting IgG production was validated using experimental data from [41] and [12]. This model incorporates constraints like media formulation, amino acid uptake rates, bioreactor dynamics, and stoichiometric balance. By imposing steady-state conditions and optimizing antibody synthesis, the model revealed a trade-off between cell growth and IgG production, reflecting biological resource allocation principles. Constraining with experimental growth rates resolved unrealistic predictions. The model generally overestimated IgG production but remained within the experimental range, performing best with the HP dataset.

Higher dilution rates can be reached in the HP simulations (Fig. 7) compared to the ones in the Late Exponential phase (Fig. 8) before having a washout. Washout happens when the dilution rate (the rate at which fresh medium is added and culture broth is removed) exceeds the specific growth rate of the cells. If the dilution rate is too high, cells are removed from the reactor faster than they can reproduce, leading to a decrease in biomass concentration.

Our model's assessment using both datasets revealed the critical influence of dilution rate and cell density on IgG production in CHO cell cultures, identifying optimal conditions at a certain dilution rate and biomass concentration. Higher IgG production rates were observed with the HP dataset, while the Late Exponential phase dataset imposed stricter constraints, and hence, the solution space was reduced in comparison to the one generated by the HP parameter values.

Identifying limiting metabolites (tyrosine, phenylalanine, threonine, valine, and notably lysine at higher rates and densities) highlighted the importance of specific amino acids in maximizing IgG synthesis. The limiting metabolite is dependent on the biomass, dilution rate, uptake rate, growth rate and IgG synthesis reaction flux. Lysine consistently emerged as a critical limiting factor as the dilution rate and biomass increased.

These insights can guide bioprocess optimization, emphasizing parameter fine-tuning and targeted nutrient supplementation to enhance IgG yields. By understanding constraints and metabolic demands, biotechnologists can develop more efficient and scalable processes for therapeutic antibody production, ultimately improving yield and reducing costs.

The study also focuses on optimizing medium composition for CHO cell cultures to maximize IgG production efficiently while minimizing costs. Utilizing computational modeling and experimental data integration, two distinct strategies were employed: economic optimization based on nutrient costs and reduction of the number of nutrients while maintaining productivity. Across different datasets (HP and Late Exponential), only 11 essential nutrients consistently supported maximum IgG synthesis, underscoring their critical roles in cellular metabolism and protein production. When a cell line is auxotrophic for a nutrient, this cell line is not able to grow in a medium lacking this element. Thus, CHO cells are auxotrophic for the 9 amino acids that are essential in mammalian cells: His, Ile, Leu, Lys, Met, Phe, Thr, Trp, and Val [43], [44], [45].

After minimizing the medium, our model predicts minimum concentrations of these amino acids, but is always different from zero. Supplementing these nutrients in the medium is necessary, and the removal of one of these from the medium would cause no growth of CHO cells at all. Our model also predicts minimum concentrations for two additional amino acids, Tyr and Cys, which are not categorized as essential, but can act as such under specific growth conditions. Furthermore, multiple CHO cell lines have been proven to be Cys auxotrophic [46]. However, several studies suggest that many CHO cell lines are also auxotrophic for proline (Pro) [47], [48] and arginine (Arg) [49]. This limitation of the iCHOv1 genome-scale model has been reported in [44], [50]. If a cell-line specific model were used instead, it would predict a different optimized medium to accommodate the additional auxotrophies.

This approach not only highlights metabolic flexibility in substituting costly nutrients with cheaper alternatives but also ensures production efficiency. The uptake rate of a metabolite depends on several factors, including the concentration of metabolites in the culture medium, the metabolic demands of the cells, the expression and activity of specific transporters on the cell membrane, and the overall cell density [51], [52]. That is why including kinetic information of the membrane transporters would be useful to improve our model.

By aligning the composition of the medium with economic considerations and bioprocessing demands, the study contributes to enhancing the sustainability and cost-effectiveness of biopharmaceutical production. Here, integrated approaches combining experimental data with FNs have proven useful for refining bioprocess strategies, offering valuable advancements in industrial biotechnology and biopharmaceutical development.

## 5 Conclusions

In this research, Flexible Nets (FNs) were exploited to integrate multiple types of biological constraints in a single model. FNs offer an adaptable modeling approach that integrates intracellular fluxes with macroscopic bioreactor dynamics, such as biomass growth and metabolite concentration. They can handle dynamic and nonlinear systems, include uncertainties, and allow for regulatory modeling based on metabolite levels. Standard methods like MFA, FBA, or dynamic FBA cannot fully capture the complexity of the system, as they focus only on intracellular fluxes. Our FN model integrates both intracellular fluxes (from FBA, MFA, etc.) and bioreactor dynamics into a single framework, creating a hybrid multi-scale approach. While it shares similarities with MFA and FBA by incorporating experimental uptake rates and solving an LPP, it uniquely includes macroscopic bioreactor variables, which standard methods cannot simulate.

The developed computational model for predicting IgG production, which was validated using experimental data from two studies, effectively incorporates constraints like media formulation, amino acid uptake rates and continuous culture dynamics as well as organisms' metabolic constraints. The model revealed a trade-off between cell growth and IgG production, which was mitigated by adjusting experimental growth rates to avoid unrealistic predictions. Although the model tends to overestimate IgG production, it remains accurate within the experimental order of magnitude, particularly with the HP dataset. This underscores the importance of including specific data to refine models.

The study highlighted specific metabolic demands, with lysine and other essential amino acids identified as limiting factors, and demonstrated that IgG production can be optimized by changing macroscopic parameters from the bioreactor such as the dilution rate and the biomass. Finally, by optimizing growth medium composition and nutrient costs, the model provides insights into enhancing IgG yields and production efficiency, demonstrating the crucial role of integrating experimental data with computational models in bioprocess optimization.

## Funding

This work was supported by the Spanish Ministry of Science and Innovation through the projects DAMOCLES-PID2020-113969RB-I00/AEI/10.13039/501100011033 and TED2021-130449B-I00, by the Aragonese Government under *Programa de Proyectos Estratégicos de Grupos de Investigación* (DisCo research group, ref. T21-23R), and by a PhD fellowship from Diputación General de Aragón (DGA) to J.L.

## CRediT authorship contribution statement

**Jorge Lázaro:** Writing – original draft, Visualization, Validation, Software, Methodology, Investigation, Formal analysis, Data curation, Conceptualization. **Teresa Joven:** Writing – original draft, Software, Methodology, Investigation, Formal analysis, Data curation, Conceptualization. **Diana Széliová:** Writing – review & editing, Validation, Supervision. **Jürgen Zanghellini:** Writing – review & editing, Validation, Supervision, Resources, Conceptualization. **Jorge Júlvez:** Writing – review & editing, Writing – original draft, Visualization, Validation, Supervision, Resources, Project administration, Investigation, Funding acquisition, Conceptualization.

## Declaration of Competing Interest

All authors declare that they have no conflicts of interest relevant to the content of this manuscript. No financial, professional, or personal relationships influenced the research presented in this paper.

## Figures and Tables

**Table 3:** IgG subunit synthesis reaction (*R*_1_ in Fig. 2). The first column contains the names of all the metabolites involved in the process. The second column refers to their molecular weights. The third column contains the stoichiometric coefficients of each metabolite.

Metabolite name	MW (*g mol*^−1^)	IgG_subunit_coef
Ala	89.09318	-39
Arg	175.2089	-14
Asn	132.11792	-26
Asp	132.09474	-23
Cys	121.15818	-16
Gln	146.1445	-30
Glu	146.12132	-31
Gly	75.0666	-44
His	155.15456	-13
Ile	131.17292	-14
Leu	131.17292	-45
Lys	147.1955	-49
Met	149.21134	-6
Phe	165.18914	-20
Pro	115.13046	-48
Ser	105.09258	-88
Thr	119.11916	-55
Trp	204.22518	-13
Tyr	181.18854	-31
Val	117.14634	-59
ATP	503.149263	-666
GTP	519.148663	-1324
*H*_2_*O*	18.01528	-1326
AMP	345.205341	664
ADP	424.177302	2
GDP	440.176702	1324
Pi	95.979301	1326
PPi	174.951262	664
H	1.00794	1326

**Table 4:** Experimental nutrient uptake rates in *mmol*$g_{DW}^{-1}$*h*^−1^ for the two datasets (HP [12] and Late Exponential phase [41]). The first row shows the experimental production of IgG, while the last row collects the experimental growth rate.

	HP	Late Exp
DM_igg[g]	2.02 ⋅ 10^−5^	0.00002437
EX_arg_L_e_	-0.01103321	-0.00664
EX_asn_L_e_	-0.074723247	-0.02168
EX_asp_L_e_	-0.025166052	-0.00345
EX_cys_L_e_	-0.00521978	-0.00227
EX_glc_e_	-0.271070111	-0.18137
EX_gln_L_e_	0.002767528	-0.03837
EX_glu_L_e_	-0.012398524	0.00257
EX_his_L_e_	-0.006457565	-0.00214
EX_ile_L_e_	-0.011808118	-0.00458
EX_leu_L_e_	-0.020184502	-0.00797
EX_lys_L_e_	-0.012583026	-0.00714
EX_met_L_e_	-0.004797048	-0.00261
EX_phe_L_e_	-0.006457565	-0.0034
EX_pro_L_e_	-0.011623616	-0.00536
EX_ser_L_e_	-0.043062731	-0.01868
EX_thr_L_e_	-0.015313653	-0.00591
EX_trp_L_e_	-0.003173432	-0.0032
EX_tyr_L_e_	-0.005276753	-0.00451
EX_val_L_e_	-0.015092251	-0.00684

**Experimental growth rate (**$h^{-1}$**)**	0.0306	0.0116

**Table 5:** Nutrient composition of the CD CHO medium according to [53].

Medium component	Concentration (mM)
Ala	0
Arg	2.07759166529374
Asn	6.02662906817444
Asp	1.35954657739258
Cys	0.379245555716144
Glc	35.1561979617665
Gln	8
Glu	1.844900322776
Gly	0
His	1.16631604999381
Ile	2.75909176846561
Lac	0
Leu	4.13863765269841
Lys	2.97227128435977
Met	0.848924084057357
NH4	0
Phe	1.31454688540469
Pro	4.71532428277354
Ser	5.16571037081442
Thr	3.0382883771534
Trp	1.0189681833183
Tyr	0.998733921520795
Val	3.08945435175835
